# Identifying neonatal hyperglycemia thresholds in preterm infants based on retinopathy of prematurity outcomes: proof-of-concept study

**DOI:** 10.3389/fped.2025.1688879

**Published:** 2025-10-15

**Authors:** Tammy Movsas, Claudia Nadernejad, Jeannette Prentice, Brooke Dudick, Amy Pribyl, Lena Sanfilippo, Brooke E. Geddie

**Affiliations:** ^1^ Research and Development Laboratory, PREEMIER LLC, Plymouth, MI, United States; ^2^ Department of Pediatrics and Human Development, College of Human Medicine, Michigan State University, Grand Rapids, MI, United States; ^3^ Neonatology, Helen DeVos Children’s Hospital, Corewell Health, Grand Rapids, MI, United States; ^4^ Department of Bioinformatics, Corewell Health, Grand Rapids, MI, United States; ^5^ Corewell Health Research Institute, Clinical Research Operations, Grand Rapids, MI, United States; ^6^ Pediatric Ophthalmology, Helen DeVos Children’s Hospital, Corewell Health, Grand Rapids, MI, United States

**Keywords:** neonatal hyperglycemia, retinopathy of prematurity, blood glucose, fetal hemoglobin, glycated hemoglobin, glycosylated fetal hemoglobin, hemoglobin A1C, neonatal outcomes

## Abstract

**Purpose:**

Neonatal hyperglycemia significantly increases risk for retinopathy of prematurity (ROP) in preterm infants, independent of other major ROP risk factors. However, glucose thresholds predictive of ROP remain undefined. This prospective study explored two glycemic metrics—mean blood glucose and glycated hemoglobin [specifically, glycated fetal hemoglobin for non-transfused infants and a neonatal-adapted hemoglobin A1C assay for transfused infants]—to identify thresholds associated with severe ROP.

**Methods:**

Infants born at gestational ages <30 weeks and hospitalized at DeVos Children's Hospital (September 2022–August 2024) were eligible. The final cohort included 98 infants. Blood glucose was monitored per clinical care, and glycated hemoglobin was measured on Day 30 using a research prototype assay. Eye exams were conducted per ROP protocol. Predictive thresholds for each biomarker were evaluated using AUC–ROC analysis.

**Results:**

Most cases of severe ROP occurred in the transfused participants, who represent the earliest gestational ages and sickest infants. Both glycated hemoglobin (A1C) and 30-day mean blood glucose were significantly higher in infants with severe ROP compared to counterparts with mild or no ROP. Both biomarkers demonstrated concordant positive predictive values of 94%. Predictive cutoffs were identified as 5.66% for A1C (*p* = 0.003) and 93.8 mg/dl for 30-day mean glucose (*p* < 0.001).

**Conclusions:**

Severe ROP may represent a clinical outcome for defining neonatal glycemic thresholds. In this pilot study, preliminary cutoffs for two independent glycemic biomarkers are lower than current NICU intervention criteria; further investigation is needed to potentially refine glucose strategies for ROP mitigation.

## Introduction

Neonatal hyperglycemia is a significant clinical dilemma in the care of preterm infants. Despite its high prevalence and established associations with increased morbidity and mortality, there remains no consensus on when to intervene (1–3). Glucose management practices vary substantially across Neonatal Intensive Care Units (NICUs), reflecting both the lack of a clearly established glycemic threshold above which adverse outcomes can be reliably anticipated and the challenge of tight glycemic control in extremely preterm infants, in whom the risk of hypoglycemia is a major concern (1–4).

This clinical ambiguity stands in contrast to the framework in adult diabetes care, where glycated (or glycosylated) hemoglobin, also known as hemoglobin A1C, is a cornerstone biomarker (5). The diagnostic threshold of A1C ≥ 6.5% in adults was not chosen arbitrarily, but rather emerged from evidence linking this level of glycemic exposure to a specific microvascular outcome—diabetic retinopathy (6, 7). This outcome-based approach allowed a transition from reactive to risk-guided care. Inspired by this precedent, we aimed to determine whether a similar outcome-based framework may help define risk thresholds for glycemic exposure in preterm neonates.

Retinopathy of prematurity (ROP) is a microvascular disorder which affects preterm infants primarily less than 30 weeks gestational age (GA). ROP shares key pathophysiologic and clinical features with diabetic retinopathy, including dysregulation of vascular endothelial growth factor (VEGF) and neovascularizaton (8–11). It is characterized by disrupted retinal vascular development, typically progressing through two distinct phases: an initial arrest of retinal angiogenesis (early stage ROP) followed by the formation of aberrant, fragile vessels in the retina, a process known as retinal neovascularization (severe, proliferative ROP) (8, 9, 12). Of particular relevance, neonatal hyperglycemia has been shown to increase risk for ROP, independent of GA, oxygen exposure and other major ROP risk factors (13–21). We therefore used ROP as a model outcome to explore whether clinically meaningful glycemic thresholds could be identified in preterm infants at high risk of ROP (GAs < 30 weeks).

To this end, we evaluated two complementary biomarkers of glycemic exposure during the first 30 days after birth: (a) the 30-day mean serum glucose and (b) a prototype glycated hemoglobin assay adapted for neonatal blood samples. Because blood transfusion can alter the glycated hemoglobin measurement, transfusion status was used to determine which assay was applied. In non-transfused infants, whose red cells predominantly contain fetal hemoglobin (HbF), we measured glycated fetal hemoglobin (Fetal GlyHb). In transfused infants, who received donor red cells containing adult hemoglobin (HbA), we quantified glycated adult hemoglobin (A1c) using a neonatal-adapted research prototype assay.

Together, mean glucose and glycated hemoglobin provide a more comprehensive assessment of neonatal glycemic burden than either measure alone. Whereas mean glucose reflects an average of point-in-time serum values, glycated hemoglobin integrates glycemic exposure continuously over time, accounting for variability and bridging the sampling gaps inherent to intermittent monitoring (22).

In this exploratory pilot study, our goal was to determine whether we can identify threshold values of each biomarker at which the risk of developing severe ROP becomes substantially elevated relative to other preterm counterparts. By doing so, we aim to lay the groundwork for outcome-based glycemic targets in preterm infants and take an initial step toward defining meaningful, risk-informed benchmarks for glucose control in the NICU

## Methods

### Blinding of investigators

All lab personnel, including the co-principal investigator (TM), were blinded to all clinical data.

### Study Population

This prospective, observational, cohort study recruited 114 neonates born between September 2022 and August 2024. Eligible participants were all infants born at gestational ages (GA) < 30 weeks and admitted to the Level IV neonatal intensive care unit (NICU) at Helen DeVos Children's Hospital, Corewell Health West. All clinical care, including nutritional support and glucose management, was provided according to the clinical judgment of the neonatology team caring for each infant. Monitoring for ROP continued until hospital discharge, retinal vascular maturation, or postmenstrual age of 42 weeks, whichever occurred first. The final study cohort included 98 infants who provided at least one fresh blood sample and survived to the study endpoint, allowing sufficient time for potential development of ROP.

### Blood Sample Collection

Blood samples were collected at designated time points. Depending upon availability, cord blood (arterial and/or venous) was obtained from residual samples collected for routine blood gas analysis. On postnatal Days 1 and 30, a drop of fresh blood was collected during routine clinical blood draws. On Day 1, samples were either capillary or arterial (depending on whether the infant had an arterial line in place), whereas the Day 30 samples were almost exclusively capillary. The number of samples collected varied across time points.

### ROP classifications

All participants, as part of clinical care, underwent serial ophthalmic examinations for ROP screening, which is standard of care for infants born at <30 weeks’ gestational age (11, 12). ROP was classified according to the International Classification of Retinopathy of Prematurity (ICROP). ROP was documented according to the ICROP (12) with ROP categorization as follows: No ROP included Stage 0 or immature vascularization. Non-proliferative ROP (NP-ROP), also referred to as Early ROP, included Stages 1 and 2. Proliferative ROP (P-ROP), also referred to as Severe ROP, included Stages 3, 4, or 5. Treatment criteria for threshold ROP was consistent with standard of care (12).

### Clinical Data Abstraction

Data were abstracted from medical records and entered into REDCap®, a secure web application configured for the study. Variables included demographics, ROP outcomes, and other clinical data.

### 30 Day mean glucose

Glucose levels were generally measured several times daily during the early postnatal period and at least once daily thereafter. The frequency of monitoring, as well as any glycemic interventions, was determined by the infant's clinical status and the judgment of the neonatology team. As part of routine clinical care, patients were managed with the goal of maintaining blood glucose concentrations within a target range of 55–150 mg/dl, whenever feasible. For each participant, 30-day mean glucose was calculated from REDCap®-extracted data. This was derived by first averaging the glucose values available for each day, and then by calculating the mean of these daily averages across the 30-day period. These levels were incorporated into the study dataset as a marker of glycemic control for statistical analyses.

### Glycated Hemoglobin Laboratory Analyses

For each blood sample, a value for glycated fetal hemoglobin or glycated adult hemoglobin (A1C) was determined dependent upon the infant's transfusion status: All samples were analyzed using Trinity Biotech platforms (Kansas City, MO): The Premier 9200 Analyzer® (boronate affinity chromatography) and The Premier Resolution Analyzer® (high-performance liquid chromatography, HPLC).

### Calculation of glycated hemoglobin metrics

#### Determination of fetal hemoglobin (HbF) and adult hemoglobin (HbA)

As part of the HPLC analysis, chromatographic peaks representing all modified and unmodified forms of fetal hemoglobin (HbF) were summed to obtain Total HbF. Similarly, peaks representing all modified and unmodified forms of adult hemoglobin (HbA) were summed to obtain Total HbA. These values representing Total HbF or Total HbA serve as denominators for calculating the percentage of glycated fetal and adult hemoglobin in each sample (as described below).

#### Glycated fetal hemoglobin (fetal GlyHb)

Fetal GlyHb was calculated by adjusting total glycated hemoglobin by Total HbF in the sample, and also by using a correction factor to account for the lower glycation rate of HbF compared with HbA. This approach assumes that all hemoglobin types in the sample have been exposed to identical glycemic conditions and is therefore valid only in the absence of transfusion (23). All cord blood and Day 1 samples were collected before transfusion, making them suitable for Fetal GlyHb assessment. For Day 30 samples, this metric was applied only when Total HbF content exceeded 80%, confirming that no transfusion had occurred.

#### Glycated adult hemoglobin (A1c)

A1C was quantified only in Day 30 samples from transfused infants in which Total HbA content exceeded 40%, the threshold required for reliable detection. A1C values were divided by the proportion of Total HbA content to account for mixed hemoglobin populations and to provide a glycation estimate specific to adult red cells.

#### Note on A1c measurement

At present, no FDA-cleared technology exists for measuring A1C in neonatal samples because of potential peak overlap between HbF and A1C on HPLC (5). The Premier Resolution Analyzer® used in this study incorporates enhanced resolution technology that has been empirically shown by the manufacturer to resolve HbF from the A1C peak, thereby mitigating this limitation. While this analyzer has received FDA certification for the measurement of HbF, it does not have FDA clearance for measurement of A1C in neonates, as the manufacturer has not sought such clearance. Accordingly, the A1C values reported here should be considered research-grade prototype estimates rather than clinical diagnostic results.

Summary of Biomarker Assignment by Sample Type:

• Fetal GlyHb: all Cord blood; all Day 1 samples; only Day 30 samples from non-transfused infants (HbF > 80%)
• A1C: only Day 30 samples from transfused infants (HbA > 40%)
• 30-Day Mean Glucose: All study participants (since blood glucose reflects a point in time value and is unaffected by transfusion)

### Statistical analyses

#### Merging data files

Lab data (blinded to clinical outcomes) were merged with REDCap clinical data to create a de-identified dataset. Analyses were conducted using SAS version 9.4 (Cary, NC).

#### Demographic and Clinical Characteristics

Given different glycated hemoglobin metrics for transfused vs. non-transfused infants, the study population was stratified by transfusion status. Demographic and clinical characteristics were assessed for the full cohort and each subgroup. Comparisons were conducted using statistical tests appropriate to variable type and distribution, including independent *t*-tests, Wilcoxon rank-sum, and Chi-square or Fisher's exact tests for categorical variables. All comparisons were pre-specified based on *a priori* clinical hypotheses, rather than being the result of exploratory data mining. Given the relatively small sample size and the targeted nature of the analyses, applying formal multiple comparison corrections could increase the risk of Type II error (false negatives) and obscure potentially meaningful associations in this hypothesis-driven study. Therefore, unadjusted *p*-values are reported to allow interpretation within the context of these predefined hypotheses.

#### Arterial vs. venous samples

Because the study samples were either capillary (a mixture of arterial and venous blood) or arterial (from an arterial line), we evaluated whether glycated hemoglobin levels varied by sample type. Matched *t*-tests were performed to compare Fetal GlyHb levels in 39 paired arterial and venous cord blood samples.

#### Comparison of day 1 fetal GlyHb levels by maternal diabetes status

Day 1 Fetal GlyHb was used as a marker of intrauterine glycemic exposure. Values were stratified by maternal diabetes status (gestational diabetes, no diabetes, type 1 diabetes, or type 2 diabetes) and compared. Day 1 Fetal GlyHb values were normally distributed by maternal diabetes status and analyzed using ANOVA.

#### Comparison of glycemic markers across ROP categories

Glycemic markers (Fetal GlyHb, 30-Day A1C, and 30-Day Mean Glucose) were analyzed across ROP categories (No ROP, NP-ROP, and P-ROP). All cord blood and Day 1 samples, drawn pre-transfusion, were analyzed for Fetal GlyHb. As previously described, 30-Day Mean Glucose was used for the entire cohort, Day 30 Fetal GlyHb was used for non-transfused infants, and A1C was used for transfused infants. Only one case of P-ROP occurred in the non-transfused group and was excluded from subgroup comparisons involving Day 30 markers. Appropriate tests (one way Anova with Tukey's *post hoc* test for pairwise comparisons, Kruskal–Wallis, Wilcoxon rank-sum, and independent *t*-tests) were used based on data distribution and pairwise comparison needs.

#### Predictive analysis of P-ROP using receiver operating characteristic (ROC) curves and area under the curve (AUC)

ROC and AUC analyses were performed to evaluate the predictive value of A1C and 30-Day Mean Glucose for P-ROP, sensitivity and specificity. Comparisons of P-ROP were made against two reference groups: (1) No ROP and (2) all others (No ROP and NP-ROP). These analyses also yielded hyperglycemic threshold values predictive of P-ROP. Optimal cutoff values were identified by selecting the point on each ROC curve that maximized the Youden index (sensitivity + specificity − 1). This approach identifies the threshold value that provides the best balance between sensitivity and specificity, thereby optimizing the biomarker's ability to distinguish infants with P-ROP from those without.

## Results

Of the 98 infants in the study cohort, 53 developed ROP. Twenty of these 53 cases were classified as severe, proliferative ROP (P-ROP); with the exception of one case, all P-ROP occurred in transfused participants. In contrast, non-proliferative ROP (NP-ROP), without progression to P-ROP, was more evenly distributed, with 17 of 33 cases in transfused infants and the remaining cases in non-transfused infants.

There were no significant differences in glycated hemoglobin levels between matched arterial and venous cord blood samples (*N* = 39 pairs, *p* = 0.66), indicating that these levels are not influenced by sample type.

Table 1 summarizes the characteristics of the overall cohort (*N* = 98) and of the transfused and non-transfused subgroups. Compared with the non-transfused subgroup, the transfused subgroup had significantly lower mean gestational age (25.0 vs. 27.9 weeks) and birthweight (752.2 vs. 1,196.5 grams). In addition to a higher proportion of infants with P-ROP, the transfused subgroup also had significantly higher rates of oxygen dependence, sepsis, and bronchopulmonary dysplasia.

**Table 1:** Demographics and characteristics of study population.

Variable	Whole population *N* = 98*	Transfused subGroup *N* = 44*	Non-transfused subgroup *N* = 49*	Subgroup comparisons: *P* value (statistical test)
Male	56%	61.3%	53.0%	0.42
Female	43.8%	38.4%	46.9%	(Chi-Square)
Black	19.5%	10.0%	25.5%	0.21
White	73.5%	82.5%	69.7%	(Fisher Exact)
Other	6.8%	7.5%	4.6%	
Non-Hispanic	90.8%	90.0%	93.0%	0.71
Hispanic	9.2%	10.0%	6.9%	(Fisher Exact)
Gestational Age (weeks)				**<0.0001**
Mean (SD)	26.6 (2.1)	25.0 (1.9)	27.9 (1.0)	(Wilcox Rank Sum)
Birthweight (grams)	976.2	752.2	1,196.5	**<0.0001** (independent *t*-test)
Mean (SD)	(336.8)	(264.4)	(257.5)	
Multiple Birth	24.4%	31.8%	20.4%	0.21 (Chi-Square)
Size for gestation age (GA)				
Small for GA	13.4%	23.2%	4.0%	**0.01** (Chi-Square)
Large for GA	13.4%	16.2%	12.2%	
Appropriate for GA	73.2%	60.4%	83.6%	
Maternal diabetes	16.6%	11.6%	23.2%	0.16 (Chi-Square)
Pre-natal steroids	87.5%	88.3%	85.4%	0.68 (Chi-Square)
Post-natal Steroids	39.8%	61.3%	20.4%	**<0.0001** (Chi-Sqr)
No ROP	45.9%	22.7%	69.3%	
Non-proliferative ROP	33.6%	38.6%	28.5%	**<0.0001**
Proliferative ROP	20.4%	38.6%	2.0%	(Chi-Square)
Intraventricular Hemorrhage	23.9%	33.3%	16.3%	0.06 (Chi-Square)
Necrotizing Enterocolitis	5.2%	9.7%	2.0%	0.17 (Fisher Exact)
Sepsis	8.5%	20.0%	0.0%	**0.001** (Fisher Exact)
Bronchopulmonay Dysplasia	41.6%	66.6%	18.3%	**<0.0001** (Chi-Square)
Pulmonary HTN	14.7%	21.9%	8.1%	0.06 (Chi-Square)
Oxygen > 28 Days	53.0%	81.8%	24.4%	**<0.0001** (Chi-Square)
%Total Fetal Hb: Day 1	94.4 [93.9, 95.0]	94.3 [93.8, 95.1]	94.5 [94.0, 95.0]	0.50 (Wilcox Rank Sum)
%Total Fetal Hb: Day 30	78.5 [30.6, 92.8]	29.9 [16.6, 38.7]	92.6 [92.1, 93.8]	**<0.0001** (Wilcox Rank Sum)

The bold values represent statistically significant results (*p* < 0.05).

* Due to missing data, the subgroup totals do not align with whole population total.

Table 2 presents the comparisons of glycemic markers measured in cord blood, on Day 1, and on Day 30. In the transfused group—where nearly all P-ROP cases occurred—both mean glycated hemoglobin (A1C) and 30-day mean glucose levels were significantly higher in infants with P-ROP compared to those with no ROP. Additionally, 30-day mean glucose levels were significantly higher in NP-ROP compared to no ROP within the transfused subgroup, a difference not observed in the non-transfused subgroup.

**Table 2:** Comparison of mean levels of glycemic markers by ROP Status at birth, Day 1 and Day 30.

Glycemic biomarker	No ROP *N* = 45^a^	Non-proliferative ROP (NP-ROP) *N* = 33^a^	Proliferative ROP (P-ROP) *N* = 20^a^	Summary of pairwise ROP comparisons: [statistical test]
Glycated Fetal Hb/Total Fetal Hb (%) Arterial Cord Blood^b^	*N* = 24 Mean: 3.0 [SD 0.1]	*N* = 13 Mean: 2.9 [SD 0.1]	*N* = 2 Mean: 2.7 [SD 0.0]	Amongst all pairs: No significant differences: *p* = 0.05 [One way Anova with Tukey's *post-hoc*]
Glycated Fetal Hb/Total Fetal Hb (%) Day 1^c^	*N* = 30 Mean: 3.0 [SD 0.2]	*N* = 22 Mean: 3.0 [SD 0.2]	*N* = 11 Mean: 3.0 [SD 0.1]	Amongst all pairs: No significant differences: *p* = 0.53 [Kruskal–Wallis (did not meet normality assumptions for ANOVA)]
Glycated Fetal Hb/Total Fetal Hb (%) Day 30	*Non-Transfused Subgroup:* *N* = 34 Mean: 3.1 [SD 0.2]	*Non-Transfused Subgroup: N* = 14 Mean: 3.1 [SD 0.1]	*Non-Transfused Subgroup: N* = 1 Mean: N/A	For No-ROP vs. NP-ROP No significant difference: *p* = 0.73 [Independent *t*-test]
Hb A1C/Total Adult Hb (%) Day 30	*Transfused* *Subgroup:* *N* = 10 Mean: 5.5 [SD 0.3]	*Transfused* *Subgroup:* *N* = 17 Mean: 5.8 [SD 0.4]	*Transfused* *Subgroup:* *N* = 17 Mean: 6.4 [SD 1.2]	**P-ROP vs. No-ROP: *p*** **<** **0.0001** No other significant pairs [Kruskal–Wallis via Dwass, Steel, Critchlow-Fligner *post-hoc* test for pairwise]
30 Day Mean Glucose (mg/dL)	*Non-Transfused Subgroup: N* = 34 Mean: 83.7 [SD 17.6] *Transfused* *Subgroup:* *N* = 10 Mean: 87.0 [SD 14.1]	*Non-Transfused Subgroup: N* = 14 Mean: 77.5 [SD 8.3] *Transfused* *Subgroup:* *N* = 17 Mean: 112.9 [SD 23.2]	*Non-Transfused Subgroup: N* = 1 Mean: N/A *Transfused* *Subgroup:* *N* = 17 Mean: 127.0 [SD 28.5]	For NP-ROP vs. No ROP: No significant differences [Wilcoxon Rank Sum test] **P-ROP vs. No-ROP:** ***P*** **<** **0.0001** **NP-ROP vs. No-ROP: *P*** **<** **0.0001** No other significant pairs [Anova with Tukey's *post-hoc* test]

The bold values represent statistically significant results (*p* < 0.05).

^a^ Variations in the number of samples collected at different time points may result in inconsistencies in the total number of samples (N) across and within columns.

^b^ Given the similarity in results between arterial and venous cord blood, only arterial results are presented.

^c^ All samples from Day 1 are pre-transfusion and are not categorized into transfused and non-transfused subgroups.

**Figure 1:**
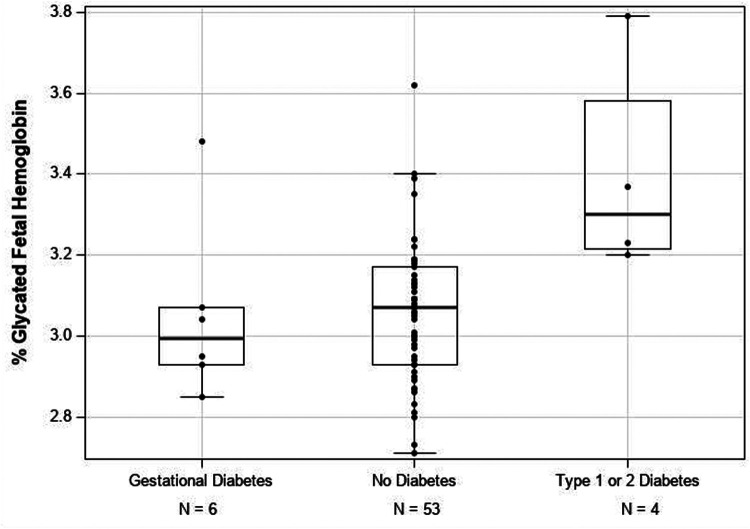
Glycated fetal hemoglobin (fetal GlyHb) levels at postnatal Day 1 in preterm infants, reflecting *in utero* glycemic control, stratified by maternal diabetes status. Infants of mothers with type 1 or type 2 diabetes were born with significantly higher Fetal GlyHb levels compared with those of mothers with gestational diabetes or no diabetes (*p* = 0.002).

Figure 1 shows that Day 1 Fetal GlyHb levels were significantly higher (*p* = 0.002) in infants born to mothers with type 1 or type 2 diabetes (mean 3.39 ± 0.27) compared with those born to mothers with gestational diabetes (mean 3.05 ± 0.22) or no diabetes (mean 3.05 ± 0.16). As seen on Figure 1, several infants born to non-diabetic mothers have Fetal GlyHb levels overlapping with the levels of infants born to mothers with type 1 or type 2 diabetes.

**Figure 2:**
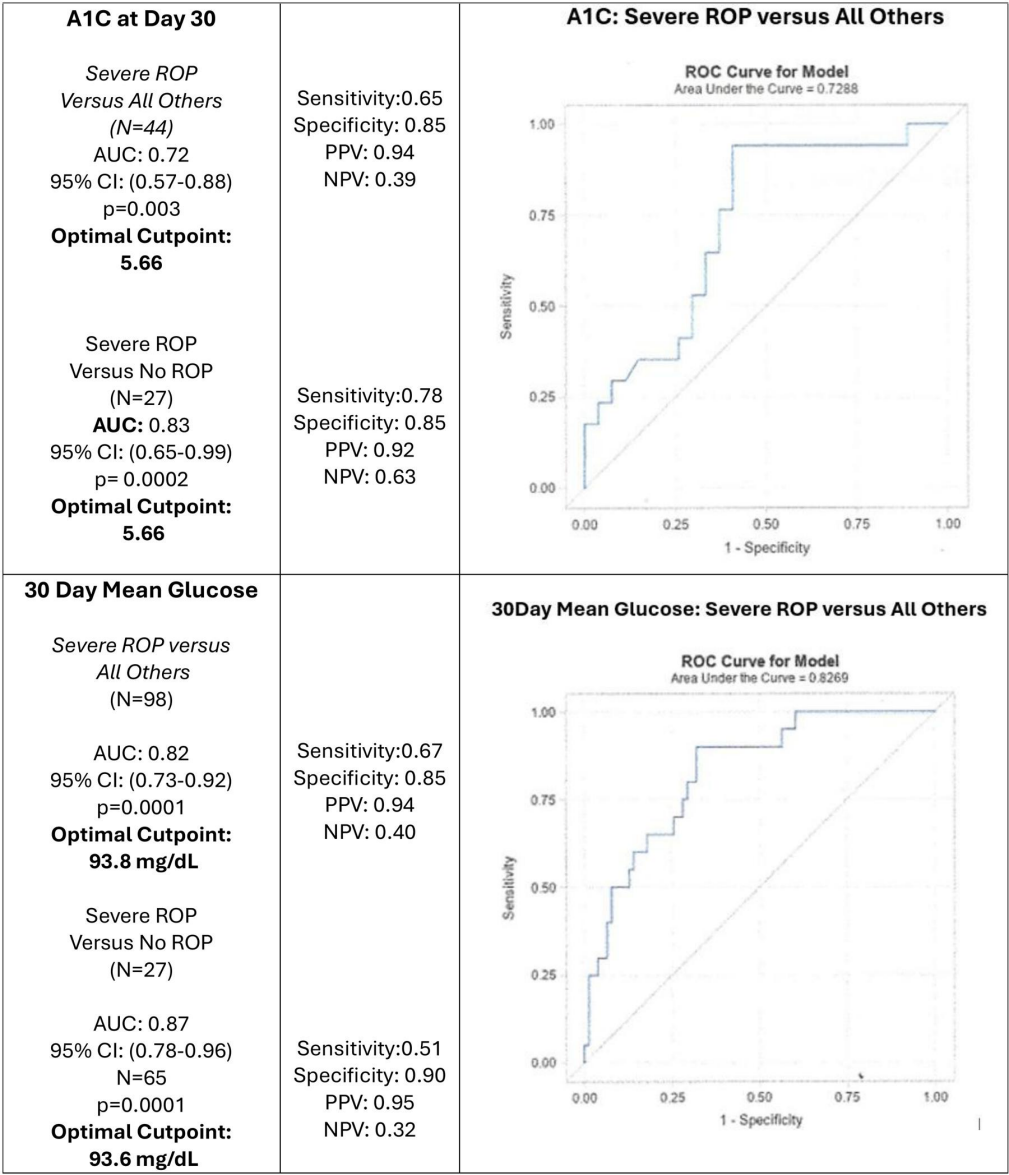
AUC–ROC analysis of hemoglobin A1C (measured at postnatal Day 30) and 30 Day mean glucose as predictors of severe ROP in preterm infants. For Hemoglobin A1C**,** the optimal cut point was 5.66, with an AUC of 0.72 (95% CI: 0.57–0.88, *p* = 0.003) for predicting severe ROP vs. all others. For 30-day mean glucose**,** the optimal cut point was 93.8 mg/dl, with an AUC of 0.82 (95% CI: 0.73–0.92, *p* < 0.0001) for predicting severe ROP vs. all others. Both biomarkers demonstrated positive predictive values of 94%.

Figure 2 shows the AUC–ROC analysis of glycemic markers as predictors of severe ROP. Both A1C and 30-day mean glucose were significant predictors. For A1C at Day 30, the optimal cut point was 5.66, with an AUC of 0.72 (95% CI: 0.57–0.88, *p* = 0.003) when comparing severe ROP to all others. For 30-day mean glucose, the optimal cut point was 93.8 mg/dl, with an AUC of 0.82 (95% CI: 0.73–0.92, *p* < 0.0001) for severe ROP vs. all others. Both biomarkers demonstrated positive predictive values of 94% and negative predictive values of approximately 40%, with corresponding sensitivity and specificity values.

## Discussion

Multiple studies have shown that neonatal hyperglycemia is significantly associated with ROP, even after adjustment for gestational age, oxygen exposure and other major ROP risk factors (13–21). In this pilot study, we explored whether ROP—particularly severe, proliferative ROP (P-ROP)—could serve as a clinical outcome for identifying glycemic levels above which the risk of severe ROP becomes significantly elevated. We focused on two distinct glycemic biomarkers: 30-day mean blood glucose and a glycated hemoglobin assay (adapted for neonatal use), measured on postnatal Day 30. Both markers, reflecting the same 30-day period, were elevated in infants who developed P-ROP compared to those who did not. For each biomarker, we identified a threshold value that predicted P-ROP with a positive predictive value (PPV) of 94%. These findings support the potential utility of these markers in guiding clinical glycemic targets for preterm infants for risk reduction.

While our study was not powered to re-affirm that hyperglycemia is an independent risk factor for ROP, the reproducibility of the PPV across two physiologically distinct biomarkers lends biological credibility to the association. Despite the known variability of neonatal glycemia (24), we observed consistent elevation in cumulative glycemic exposure among infants who developed P-ROP. Mean glucose captures discrete fluctuations and acute spikes, whereas glycated hemoglobin offers an integrated biochemical record over time. That both measures converged on similar predictive thresholds suggest the signal is unlikely to be a statistical artifact or assay-specific anomaly, but more likely reflects an underlying biological association.

Our findings highlight the need of establishing evidence-based thresholds for neonatal hyperglycemia, which remains an inconsistently managed clinical domain (1–3). In full-term newborns, glucose levels rarely exceed 126 mg/dl, yet in NICU settings, clinical intervention is typically deferred until levels reach 150–180 mg/dl (1–3). The predictive thresholds identified in our study—94 mg/dl for mean glucose and an A1C of 5.66 (corresponding to an estimated average glucose of ∼114 mg/dl)—fall well below these conventional cutoffs. While this study does not establish causal harm at a particular level, the findings raise the possibility that even moderate, sustained hyperglycemia may be biologically meaningful, particularly in the context of the metabolically immature and oxidatively vulnerable retina of preterm infants.

The threshold of 94 mg/dl for 30-day mean glucose identified in our study is consistent with a study reporting median glucose levels of 95 mg/dl in their ROP group (16). Our findings also support a study where sustained glucose levels above 126 mg/dl for more than nine days were predictive of P-ROP (14), a threshold not commonly used to initiate treatment. Additionally, our results offer insights into a low birthweight study involving over 20,000 infants which did not establish a link between hyperglycemia and P-ROP (25). The study defined hyperglycemia as above 180 mg/dl which may have caused misclassification of many of their study participants as non-hyperglycemic, even though their levels were significantly above physiological norms.

Hyperglycemia as a risk domain for P-ROP is closely aligned with ROP's underlying pathophysiology. Dysregulation of vascular endothelial growth factor (VEGF) and heightened oxidative stress are central mechanisms driving the neovascularization characteristic of P-ROP (11, 26–28). Of particular relevance, hyperglycemia is known to exacerbate both processes—by intensifying oxidative injury and upregulating VEGF expression, as well documented in diabetic retinopathy (10, 26, 27). The strong predictive performance of our glycemic biomarkers, together with the biological plausibility, suggests that hyperglycemia may, in some infants, act as a driver of VEGF dysregulation or, alternatively, may amplify disease progression in the presence of other concurrent risk factors.

Conversely, the negative predictive values (NPVs) of both biomarkers are approximately 40%. This large disparity between high PPV and low NPV implies that while the presence of sustained hyperglycemia signals a markedly increased risk for severe disease, P-ROP still develops in its absence. This aligns with the oxygen-driven model of ROP pathogenesis, which involves hyperoxia with subsequent hypoxia, which, like hyperglycemia, leads to VEGF dysregulation and neovascularization (8, 9, 12). The discrepancy between PPV and NPV reinforces the notion that ROP is a heterogeneous condition with potentially multiple VEGF-driven pathways.

Though this study does not provide evidence of a direct causal relationship, these findings suggest that hyperglycemia may represent one of several interrelated biochemical markers or risk endotypes within the multifactorial spectrum of ROP—where glycemic-targeted interventions might be effective for some infants—while others may develop the disease through the well-established oxygen pathway, mixed VEGF-mediated mechanisms or via an alternative pathway.

The biomarkers in this study reflect either cumulative or average glycemic exposure over a 30-day period rather than short-term fluctuations within that timeframe. Notably, some prior studies have suggested that glycemic variability itself may be a significant risk factor for severe ROP (24, 29). This highlights the need for further investigation that considers both cumulative glycemic exposure (as done in this study) and glycemic variability.

Our study subgroups were classified based on transfusion status to align participants with the glycemic metrics used for the Day 30 blood samples: A1C for transfused newborns and Glycated Fetal Hemoglobin (Fetal GlyHb) for non-transfused infants. In general, preterm infants who meet the criteria for transfusion are more severely ill compared to their non-transfused counterparts (30). Consequently, the transfused group exhibited a higher prevalence of ROP risk factors, including lower GAs, increased O_2_ dependence, and reduced fetal hemoglobin due to transfusions. The lower GAs in the transfusion subgroup also imply a greater degree of metabolic immaturity, which affects glucose regulation and oxidative stress management. Except for one case, P-ROP occurred exclusively in the transfused group.

In addition to gestational age, multiple clinical factors may have influenced glycemic levels in our study population. These may include nutritional support (parenteral and enteral), medications (such as postnatal steroids), glycemic interventions (such as insulin administration), intercurrent illnesses (such as sepsis), and fluid or glucose infusion practices. The effects of these variables on glucose homeostasis were not within the scope of the present analysis. However, future investigations explicitly designed to examine the interplay of these factors will be important for advancing our understanding of glycemic regulation in preterm infants.

The rate of ROP, including P-ROP, is higher in our overall cohort compared with published incidences among screened preterm infants worldwide (31, 32). This likely reflects the preterm infant population at Helen DeVos Children's Hospital (HDVCH), where this study was conducted. HDVCH has a Level IV NICU with a special unit for extremely premature infants and therefore receives transfers of such infants from surrounding hospitals. Consequently, the NICU has a disproportionately high number of very low gestational age (GA) infants who are at increased risk for P-ROP. In our study, nearly all P-ROP cases occurred in the transfused subgroup, which had a mean GA of 25.0 weeks. Within this subgroup, the incidence of any ROP and P-ROP was 77.2% and 38.6%, respectively. These rates are lower than those reported in a study of over 2,000 preterm infants, in which infants born at GAs ≤25.0 weeks had rates of 92.9% for any ROP and 64.3% for severe ROP (31).

Our transfusion-specific methodology helped mitigate the confounding effects transfusions can have on glycated hemoglobin measurements, a critical consideration given the high frequency of transfusions in very preterm infants (16, 33). In this pilot study, over 40% of infants received transfusions. The absence of transfusion stratification may explain why a prior study using glycated albumin—another marker of chronic glycemia—failed to detect an association with ROP, despite higher blood glucose levels in affected infants (16). Without accounting for transfusion status, the relationship between chronic glycemia and disease may have been obscured. In contrast, our approach allowed for more precise biomarker assignment, enhanced signal resolution, and inclusion of transfused infants—the sickest and most at-risk subgroup.

Because risk factors for mild, non-proliferative ROP (NP-ROP) may differ from those for proliferative ROP (P-ROP) (34, 35), we examined the biomarkers for NP-ROP separately from P-ROP. Among non-transfused infants with NP-ROP, we found no evidence of hyperglycemia. In contrast, transfused infants with NP-ROP exhibited significantly higher 30 day mean glucose levels compared to those without ROP. This pattern raises the possibility that hyperglycemia may contribute to early ROP progression or reflect a distinct disease trajectory in more critically ill infants, warranting further investigation in a larger cohort.

All Day 1 samples were obtained prior to any transfusions, allowing the use of glycated fetal hemoglobin (Fetal GlyHb) as a marker of glycemic exposure. Because Fetal GlyHb reflects glucose levels accumulated over the preceding weeks, Day 1 values provide insight into the infant's *in utero* glycemic environment. In this study, Day 1 Fetal GlyHb was significantly higher in infants born to mothers with pre-existing diabetes (type 1 or type 2) compared with those born to mothers with gestational diabetes or no diabetes. This finding is noteworthy because exposure to maternal hyperglycemia during pregnancy is known to influence offspring health in both the short and long term (3). Importantly, most prior human data on fetal glucose exposure has been inferred indirectly through maternal indicators such as mother's hemoglobin A1C (3). By contrast, our study demonstrates elevated glycated fetal hemoglobin directly from the infant's own blood—a more specific measure of infant's glucose control. However, because only four mothers in our cohort had type 1 or type 2 diabetes, the study was underpowered to assess whether these elevations were associated with differences in ROP risk.

Study data collection was not continued after hospital discharge, so a few NP-ROP cases may have progressed to P-ROP after follow-up ended. While such misclassification may have slightly increased the number of P-ROP cases, it is unlikely to have significantly altered our findings. Another limitation is the absence of baseline A1C values in donor blood used for transfusions, which may have influenced the transfused A1C results. Additionally, the shorter half-life of transfused adult erythrocytes could have affected A1C measurements (36). Nonetheless, the strength of the A1C signal—despite these constraints—demonstrates the magnitude of glycemic differences in infants who developed P-ROP compared to the others.

The single-center design of this study may limit generalizability. Additionally, as noted earlier, this study was not powered for multivariate analysis. However, multiple studies have identified hyperglycemia as a significant, independent risk factor for ROP (13–21), These prior studies include case-control studies, multicenter cohorts, and prospective and retrospective analyses which employed robust multivariate modeling (13–21). Moreover, the biological plausibility of hyperglycemia's role in proliferative ROP is well supported: dysregulated VEGF is a central driver of the disease, and hyperglycemia is known to dysregulate retinal VEGF (8–10, 37, 38).

## Conclusions

This proof-of-concept study suggests that ROP—particularly its advanced, proliferative form—may serve as a clinically meaningful outcome for establishing glycemic thresholds in preterm infants. The preliminary predictive cutoffs identified for both neonatal A1C and 30-day mean blood glucose are substantially lower than the values that typically prompt clinical intervention in the NICU. These findings underscore the need for evaluation of these thresholds in multicenter cohorts and to determine whether refined glucose management may mitigate ROP.

## Data Availability

The datasets presented in this article are not readily available because Data Sharing Statement: Because this preterm infant study was conducted at a single institution during a defined time period, the dataset cannot be shared in order to completely protect the neonates’ confidentiality. In addition, the parental consent process did not authorize data deposition of the infant participants into a repository or the sharing of data with other researchers. Therefore, in accordance with the approved institutional review board protocol and applicable data protection regulations, the dataset cannot be shared outside the study team. Requests to access the datasets should be directed to We are not able to accept requests for data sharing.
